# Importance of telemedicine in mild cognitive impairment and Alzheimer disease patients population during admission to emergency departments with COVID-19

**DOI:** 10.1097/MD.0000000000032934

**Published:** 2023-02-22

**Authors:** Francesco Corallo, Giuseppa Maresca, Lilla Bonanno, Viviana Lo Buono, Jolanda De Caro, Carmen Bonanno, Caterina Formica, Angelo Quartarone, Maria Cristina De Cola

**Affiliations:** a IRCCS Centro Neurolesi Bonino-Pulejo, Messina, Sicily, Italy.

**Keywords:** COVID-19, dementia, telehealth, telemedicine

## Abstract

In March 2020, the World Health Organization declared a global pandemic due to the new coronavirus SARS-CoV-2, and several governments have planned a national quarantine to control the spread of the virus. Acute psychological effects during hospitalization in frail elderly individuals with special needs, such as patients with dementia, have been little studied. The greatest distress manifested by these kinds of patients was isolation from their families during hospitalization. Thus, structured video call interventions were carried out to family caregivers of patients diagnosed with dementia during their hospitalization in the COVID-19 ward. The purpose of this quasi-experimental study was to assess changes in cognitive and behavioral symptoms in both patients and caregivers. All study participants underwent psychological assessments. Specifically, the psychological well-being states of patients and their caregivers were measured at admission (*T*0) and discharge (*T*1) using psychometric tests and clinical scales. Each participant received an electronic device to access video calls in addition meetings were scheduled with the psychologist and medical team to keep caregivers updated on the health status of their relatives. A psychological support and cognitive rehabilitation service was also provided. Significant differences were found in all clinical variables of the caregiver group. Results showed a significant relationship in the quality of life score between the patient and caregiver groups. The results of this study has highlighted the importance of maintaining significantly effective relationships during the hospitalization period of patients admitted to COVID wards.

## 1. Introduction

The epidemic of COVID-19 has had a profound impact on the organization of clinical and social care activities for people with dementia and their caregivers. Undoubtedly, preventive measures to limit the spread of COVID-19 are essential, but in patients with dementia, they have often caused social isolation, and exacerbated behavioral and psychological symptoms of dementia (BPSD)^[1,2]^ resulting in increased caregiver burden.^[3,4]^ BPSD, also known as neuropsychiatric symptoms, represent a heterogeneous group of noncognitive symptoms, and behaviors that occur in people with dementia. BPSDs include agitation, aberrant motor behavior, anxiety, euphoria, irritability, depression, apathy, disinhibition, delusions, hallucinations, and altered sleep or appetite.^[5]^ For this reason, we have witnessed the development of several remote interventions aimed at the dementia patient-caregiver couple, implemented through the use of digital tools with telephone conversations, video calls (using smartphones, PCs, tablets), and other communication channels as e-mail.^[6,7]^ Although remote, these interventions have made it possible to monitor the clinical conditions of people with dementia, support caregivers in disease management, identify risk conditions, ensure care, and continuity of care through continuous communication with the care network.^[8–10]^ Thus, remote interventions have yielded great benefits as reduction of care costs and travel time, representing a viable alternative when the physical presence of the user/family member is prevented and/or in emergency situations.^[11,12]^ These interventions can provide an opportunity to improve and simplify the care process and promote continuity of care although have important limitations, such as the difficulty in establishing a relationship with the other person, the need for the dementia patient to be assisted in using the technology, and the need to learn the computer program to participate in treatment.^[13]^ Patients hospitalized for COVID-19 infection face a high mortality risk. Thus, patients families may face possible impending bereavement without being able to see their loved 1.^[14–16]^ The stressful condition resulting from worrying about the death of a loved 1 is considered the most stressful event among those that make up the normal human experience.^[17–20]^ Indeed, for both COVID-19 patients and their family members, not knowing how the disease will progress and having to abide by the rules of the inpatient facility can be a source of great anxiety.^[21–23]^ Hospitals and other health care facilities have to limit or prohibit the physical presence of visitors, in order to control the spread of the virus within these facilities. Such restrictions have induced new stressors for patients and caregivers, making it necessary for hospitals to implement procedures to support the families of patient hospitalized for COVID-19 infection, with particular regard to those fragile populations such as people with dementia.^[24]^ In several European countries, including Italy, a high proportion of deaths from COVID-19 (approximately 20%) have occurred among people with dementia, probably due to the difficulty in adhering to health and safety regulations, as well as the concomitant presence of other chronic diseases. In addition, approximately 20% of persons with dementia live in residential facilities where virus circulation is more massive.^[25,26]^ In light of scientific evidence, we hypothesized that establishing a contact between dementia inpatients and their families via video conferencing could minimize the possible negative impact of social distancing measures necessitated by the COVID-19 pandemic.^[26]^ Main purpose of this study is to report changes on cognitive and behavioral symptoms of patients with dementia and on caregiver stress conditions after this intervention.

## 2. Materials and methods

### 2.1. Study design and setting

This was a quasi-experimental study; 28 patients (with mild to moderate neurocognitive impairment) and their caregivers were recruited. The study protocol was approved by the Local Ethics Committee according to the 1964 Declaration of Helsinki and its later amendments. The PI of the study and his co-investigators contacted caregivers by telephone and, after signing the informed consent by email, tests and clinical scales were administered at patient admission (*T*0) and before discharge (*T*1), that is, about 25 days after. The study was developed within a low-intensity Covid ward. The patients recovery from the infection was steady, clinical conditions were not serious, and admission to the COVID-19 ward was a precautionary measure. All patients enrolled in the study did not present alterations in consciousness either at admission or during hospitalization. They manifested homogeneous/common signs and symptoms, including fever (TC > 37.5; < 39.5), dry cough, mild to moderate dyspnea, generalized asthenia, and myalgias. Likely, the sample was found to have SarsCoV2 alpha variant B.1.1.7, first identified in UK on Dec. 14, 2020. The video calls were taken through a specific telematic platform installed on the institute’s network. The subjects underwent daily psychological interviews in which the psychologist investigated the caregiver’s burden about following the clinical condition remotely. The device was also used for videoconferencing meetings with relatives, always followed with the support of dedicated staff. Caregivers could also use this service to carry out daily teleconsulting with the medical equip who updated the relatives about their health condition. The sessions were structured as follows: the duration was about an hour, the video calls took place within the ward, always in the presence of the psychologist. Initially, the relatives had the possibility of dealing with the doctors of the ward who provided information about the relative health conditions. The device was used at the patient bed for meetings with family members and at the end, the psychologist held a private meeting with the caregiver in which could talk about his concerns regarding the patient clinical condition and also discuss the caregiver’s burden respect to the management at the back home.

The study was divided into 2 phases.

### 2.2. Phase 1 – enrollment

Subjects were evaluated at hospital admission (*T*0) through an objective and neurological examination by the physician and cognitive assessment by the psychologist: in order to confirm a neuro-cognitive profile compatible with dementia. In particular, cognitive evaluation was conducted with patients face to face. Functional evaluation, affective and behavioral assessment were conducted with caregivers of relatives hospitalized through telemedicine platform.

#### 2.2.1. Functional status evaluation.

● Functional indipendence measure is a scale for measuring disability. the questionnaire described 18 activities of daily living (13 motor-sphincteric, 5 cognitive). the scores ranging from 1 (complete dependence on others) to 7 (complete self-sufficiency). The cumulative scores produce a quantitative index of the patient disability.^[27]^
● Clinical dementia rating scale is for the assessment of dementia severity in elderly subjects. The CDR is a 5-point scale used to characterize 6 domains: memory, orientation, judgment, and problem solving, business, home and hobbies, and personal care. The score is the result of the patient evaluation and the interview with the caregiver.^[28]^

#### 2.2.2. Assessment of affective and behavioral status.

● Neuropsychiatric inventory questionnaire is an informant-based interview that assesses neuropsychiatric symptoms of the participant over the previous month. the severity of the symptoms present within the last month on a 3-point scale (1 = mild, 2 = moderate, 3 = severe).^[29]^
● Hamilton anxiety rating scale; The HAM-A was one of the first rating scales developed to measure the severity of anxiety symptoms, The scale consists of 14 items, each defined by a series of symptoms, and measures both psychic anxiety (mental agitation and psychological distress) and somatic anxiety (physical complaints related to anxiety).^[30]^
● Geriatric depression scale to evaluate depressive symptoms in elderly patients. Scores of 0-4 are considered normal, depending on age, education, and complaints; 5 to 8 indicate mild depression; 9 to 11 indicate moderate depression; and 12 to 15 indicate severe depression.^[31]^
● EQ-5D EQ-5D is an instrument which evaluates the generic quality of life (QOL) developed in Europe and widely used. The EQ-5D descriptive system is a preference-based HRQL measure with 1 question for each of the 5 dimensions that include mobility, self-care, usual activities, pain/discomfort, and anxiety/depression.^[32]^
● Neuropsychological evaluation of patients
● Mini mentali state examination (MMSE) is for the assessment of general cognitive profile. the maximum score for the MMSE is 30. A score of 25 or higher is classed as normal. If the score is below 24, the result is usually considered to be abnormal, indicating possible cognitive impairment.^[33]^

#### 2.2.3. Caregivers emotional assessment.

● Zarit burden inventory is an interview used for the evaluation of the caregiver burden of a family member with chronic or degenerative pathologies. consists of 22 items. scores are assigned by a Likert scale from 0 (never) to 4 (almost always). The items investigate how the patient disability impacts on the QOL, suffering psychological, guilt, financial difficulties, shame and social and family difficulties of caregivers.^[34]^
● Beck depression inventory. is a 21-item, multiple-choice inventory to assess presence of depressive symptoms. Total score of 0 to 13 is considered minimal range, 14 to 19 is mild, 20 to 28 is moderate, and 29 to 63 is severe depressive condition.^[35]^

### 2.3. Phase 2

Subjects underwent daily psychological interviewing and then met via video conferencing with relatives. Each session was 1 hour in duration; the video calls took place within the ward. At the end of hospitalization patients were submitted to final evaluation with the same tests used at baseline (*T*0) in face-to-face modality. While caregivers were submitted to tests with through telemedicine platform.

### 2.4. Patient inclusion criteria

● Positivity to molecular testing for COVID-19.
● Diagnosis of dementia.
● Informed consent signed by patient or support administrator.
● Degree of dementia from severe to moderate: MMSE > 12 (range 13–16).

### 2.5. Patient exclusion criteria

● Absence of supervision.
● Failure to sign informed consent.
● Severe primary progressive aphasia.

### 2.6. Caregiver inclusion criteria

● Greater than 18 years of age.
● Signature of informed consent.

### 2.7. Evolution of general clinical conditions of the study population

None of the selected patients had episodes of acute respiratory failure and none required urgent clinical intervention. We enrolled 21 patients with Alzheimer disease diagnosed for 10 years and 7 patients diagnosed as Mild Cognitive Impairment for 2 years. For the treatment of acute COVID-19 infection, the therapy administered was the same for all patients, based on the combination of dexamethasone 6 mg (as a single intravenous administration) for 10 days and anticoagulant therapy with low molecular weight heparins. Dose adjustments or a different choice of the active ingredient of low molecular weight heparins, between enoxaparin 4000 IU/L and fondaparinux 2.5 mg, were made on the basis of the patient general clinical characteristics, particularly in relation to renal function values. In addition, all patients underwent an average of at least 6 days of noninvasive low-flow oxygen therapy.

All patients selected for the study underwent daily nutrition monitoring by blood gas analysis. In addition, the cohort of patients taken into consideration, following an epidemic outbreak developed in an old people’s home, consisted of patients defined as “frail” due to the following characteristics: suffering from chronic diseases, presence of comorbidities such as hypertension, diabetes, cerebral and cardiovascular diseases, all these diseases were specific pharmacological treated, clinical instability, and reduced self-sufficiency; in some cases, social and family problems may be added.

### 2.8. Statistical analysis

The sample analysis was conducted on 2 groups: patients and caregivers. A nonparametric analysis was carried out because the results of the Shapiro normality test indicated that most of the target variables were not normally distributed. Thus, numerical data were presented as median, and first-third quartile and the Mann–Whitney *U* test was used. Finally, we performed an interaction effect analysis (improved time) by calculating the *T*1 to *T*0 differences in variables scores to Spearman correlation analysis. Analyses were performed using an open source R3.0 software package. A 95% of confidence level was set with a 5% alpha error. Statistical significance was set at *P* < .05.

## 3. Results

**Figure 1.**
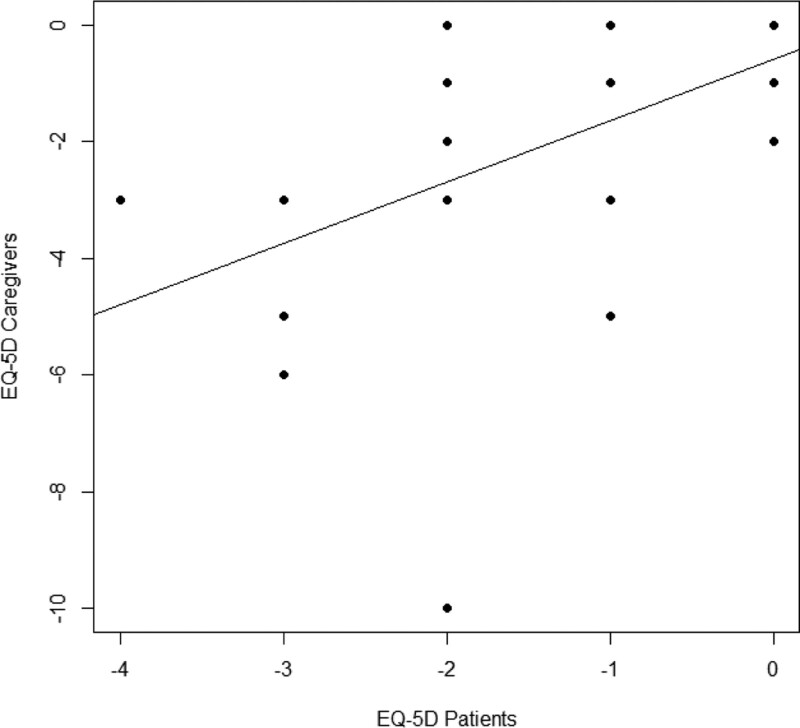
Correlation between EQ-5D score in patient and caregiver groups.

In Table 1, demographic and clinical characteristics of groups are reported. Mann–Whitney *U* test showed significant differences (*P* < .05), in patient group, in almost all clinical variables (Table 2) except in clinical dementia rating scale that remained constant between *T*0 and *T*1. In the caregivers group significant differences in all clinical variables (*P* < .05) were found. Spearman correlation analysis showed a significant relationship in EQ-5D score between patient and caregiver groups (*R* = 0.52; *P* = .002) (Fig. 1).

**Table 1:** Clinical and demographic characteristics of groups.

	Patients	Caregiver
	Mean ± SD	Mean ± SD
Age	83.68 ± 10.16	51.71 ± 9.51
Gender		
Male	5 (17.86%)	12 (42.86%)
Female	23 (82.14%)	16 (57.14%)
Education	6.25 ± 3.37	12.89 ± 3.60
Type of caregiver		
Son	-	7 (25%)
Daughter	-	15 (54%)
Husband	-	5 (18%)
Wife	-	1 (4%)
Time between *T*O and *T*1	24.28 ± 2.69	24.28 ± 2.69
Diagnosis		
AD	21 (75%)	-
MCI	7 (25%)	-

AD = Alzheimer disease, MCI = mild cognitive impairment, SD = standar deviation.

**Table 2:** Clinical scores before and after treatment of groups.

	*T*0	*T*1	*P* value
	Median (I–III quartile)	Median (I–III quartile)	
*Patients group*			
FIM	54.0 (54.0–81.25)	60.0 (54.0–85.0)	.01
CDRS	2.0 (1.0–2.0)	2.0 (1.0–2.0)	-
NPI	28.0 (7.5–60.0)	28.0 (6.0–51.0)	.003
HRS_A	18.0 (14.5–22.0)	15.0 (12.0–18.5)	< .001
GDS	20.0 (18.0–21.5)	18.0 (14.0–20.0)	< .001
EQ-5D	12.5 (9.0–14.0)	10.5 (7.75–13.25)	< .001
MMSE	12.5 (8.0–21.25)	13.45 (8.35–22.57)	< .001
*Caregivers group*			
ZIB	57.0 (51.7–60.0)	47.0 (46.0–54.0)	< .001
EQ-5D	7.0 (4.0–9.0)	4.0 (4.0–5.2)	< .001
HRS	13.0 (8.0–16.5)	10.0 (8.0–11.2)	< .001
BDI	12.0 (9.0–18.0)	9.0 (8.0–11.0)	< .001

BDI = beck depression inventory, CDRS = clinical dementia rating scale, FIM = functional indipendence measure, GDS = geriatric depression scale, MMSE = mini mentali state examination.

## 4. Discussion

Dementia affects more than 55 million patients worldwide, with significant social, economic and psychological impact. However, many patients with Alzheimer disease and other related dementias have limited access to effective and personalized care. The pandemic of COVID-19 has accelerated the use of telemedicine, which has promising potential to address this important gap. Angelopoulou et al^[36]^ (2022), in a review, analyze how telemedicine can improve the quality of care for such patients, concluding that telemedicine can significantly improve all aspects of quality of care for patients with dementia. The restrictive measures introduced to contain COVID-19 infection provided a useful opportunity to adopt and concretely test the use of telemedicine interventions in the care-taking process and continuity of care. During the first months of the national lockdown, adopting remote interventions was a necessity in order to adequately respond to the needs and requirements of a disoriented, frightened and distraught population and to stem, as far as possible, the sense of loneliness and abandonment.^[37]^ In such a critical historical moment, relatives perceived increased caregiver’s burden combined to the fear of contagion and the effects of social isolation. Like other long-distance realities, the “Let Keep Active” project has provided continuous support and concrete help to caregivers and people with dementia in everyday life. Through specially ad hoc activities, caregivers were given the opportunity and the tools to share pleasant and entertaining moments together avoiding dysfunctional dynamics. At the same time, for people with dementia a new daily routine was structured that recalled the activities of the day center attended before the lockdown.

The aim of this study was to observe whether the use of remotely technology during isolation in COVID-19 unit could contribute to improve QOL of elderly patients and their caregivers. Our results are very encouraging. We observed an improvement in mental health status during hospitalization and this result seems to be due to this type of telemedicine intervention in both groups caregivers and demented patients. It is important to point out that in our sample all patients were from a nursing home and therefore the inpatient setting was similar to their home. The objective of this study was not focused on the patient cognitive impairment, rather to improve the relationship between the hospitalized patient and caregiver that affects the psychological well-being and QOL of both groups. As the telemedicine system has allowed caregivers to always updated on the mental and physical health of family members. We hypothesized that through the use of telemedicine systems, it is possible to conduct support activities for patients with neuro-cognitive disorders in the wards of COVID-19 and, indirectly, for their caregivers.

Caregiver burden is recognized as a social and scientific phenomenon. Several studies showed that caregivers suffered anxiety and depressive symptoms during the hospitalization of their relatives.^[38–42]^ We found an increased prevalence of symptoms of anxiety, feelings of helplessness, and distress reported by caregivers. during the course of relatives disease. Communication through systems remotely allowed the involvement not only of verbal communication, but also non-verbal communication and facial mimicry, a method to bypass face-to-face meetings allowing a satisfactory relationship even at a distance. The support of psychologists during these procedures it became useful to support new ideas, thoughts about elderly people with dementia and their relatives in a perspective of self-preservation and reduction of burden on caregivers in order to promote a coexistence and avoid leading to situations of great discord. These results are in line with other studies emphasizing that a tele psychotherapeutic approach improved psychological symptoms, with a significant reduction in patients anxiety and depression.^[43]^ These results were supported by a study conducted between March and May 2020 by Lai and colleagues (2020) in which the impact of telecare on caregivers of people with cognitive decline, during the COVID-19 pandemic, was evaluated. This research was conducted with 60 pairs of caregivers and people with dementia enrolled from a day care center. These findings suggested that teleassistance interventions, through the videoconference, had provided support to the caregiver in the management of their relatives and this method had greater benefits than telephone interviews alone.^[44]^

Telecare interventions had a good impact on the caregiver’s QOL and also on the maintenance of the cognitive level of the family members suffering from cognitive decline.^[45]^ These results suggested the possibility that remote interventions could be used in common practice in the future to support the relationship between caregiver and family member and, at the same time, ensure therapeutic continuity-assisted care.^[46]^ Although literature studies confirmed the validity and reliability of telemedicine in the diagnosis and follow-up of patients with dementia.

The limitations of our study was the lack of data regarding the benefits obtained during the COVID-19 pandemic,^[47,48]^ The age of the caregiver the successfulness of the telerehabilitation, but in our group the age distribution is homogeneous.

The lack of a control group to compare the results obtained, the small number of participants and the lack of accurate sociodemographic and socioeconomic information of caregivers. A multicenter study with larger groups of patients and caregivers is needed to confirm these preliminary data. Moreover, it could be interesting to evaluate caregivers satisfaction concerning the usability and feasibility of telemedicine platforms.

## 5. Conclusion

To dates, the coronavirus pandemic is still a worldwide health emergency. This momentous event has disrupted lives and had a profound impact on the organization of clinical and social care activities for people with dementia and their caregivers remote interventions, therefore, could be a viable alternative in the future in cases where cases when in-presence activities are not possible, such as the outbreak of a pandemic or when the physical presence of the user or family member is prevented for any reason (e.g., fear of contagion for the resumption of activities).

## Author contributions

**Conceptualization:** Francesco Corallo, Giuseppa Maresca.

**Data curation:** Lilla Bonanno, Maria Cristina De Cola.

**Formal analysis:** Maria Cristina De Cola.

**Methodology:** Viviana Lo Buono.

**Project administration:** Jolanda De Caro.

**Supervision:** Giuseppa Maresca.

**Visualization:** Carmen Bonanno.

**Validation:** Angelo Quartarone.

**Writing – review & editing:** Caterina Formica.
